# Combination therapy with capecitabine and temozolomide in patients with low and high grade neuroendocrine tumors, with an exploratory analysis of O^6^-methylguanine DNA methyltransferase as a biomarker for response

**DOI:** 10.18632/oncotarget.22001

**Published:** 2017-10-24

**Authors:** Dwight H. Owen, Andrew J. Alexander, Bhavana Konda, Lai Wei, Jessica A. Hemminger, Carl R. Schmidt, Sherif R.Z. Abdel-Misih, Mary E. Dillhoff, Jennifer A. Sipos, Lawrence S. Kirschner, Manisha H. Shah

**Affiliations:** ^1^ Division of Medical Oncology, Department of Internal Medicine, The Ohio State University Comprehensive Cancer Center, Columbus, OH, USA; ^2^ Division of Infectious Disease, Department of Internal Medicine, The Ohio State University Comprehensive Cancer Center, Columbus, OH, USA; ^3^ Center for Biostatistics, The Ohio State University Comprehensive Cancer Center, Columbus, OH, USA; ^4^ Department of Pathology, The Ohio State University Comprehensive Cancer Center, Columbus, OH, USA; ^5^ Division of Surgical Oncology, Department of Surgery, The Ohio State University Comprehensive Cancer Center, Columbus, OH, USA; ^6^ Division of Endocrinology, Diabetes & Metabolism, Department of Internal Medicine, The Ohio State University Comprehensive Cancer Center, Columbus, OH, USA

**Keywords:** neuroendocrine tumors, MGMT, temozolomide, capecitabine, immunohistochemistry

## Abstract

Recent advances in the treatment of neuroendocrine tumors (NET), including the combination regimen of capecitabine and temozolomide (CAPTEM), have mostly focused on grade 1 and 2 pancreatic neuroendocrine tumors (pNET). We undertook a retrospective review of 38 patients with advanced NET treated with CAPTEM, including patients with non-pancreatic tumors as well as grade 2 and 3 tumors. O^6^-methylguanine DNA methyltransferase (MGMT) expression was assessed as a predictive biomarker. We found that CAPTEM demonstrated activity in patients with all grades and in pNET and non-pNET. Median progression free survival (mPFS) was 13.0 months (95% CI: 5.6-17.0) and median overall survival (mOS) 29.3 months (95% CI 17.7 - 45.3). Among evaluable patients, there were 11 (38%) partial responses, 15 (52%) stable disease, and 3 (10%) progressive disease for a disease control rate of 90%. A higher rate of partial responses occurred in patients whose tumors had low levels of MGMT expression (63%) compared to intermediate-high (17%) (p=0.19). Our results show that CAPTEM therapy is active in patients with NET including in previously treated patients and in those with poorly-differentiated histology. We observed a trend towards increased response rate, median PFS, and median OS in patients whose tumors had low MGMT protein expression.

## INTRODUCTION

Neuroendocrine tumors (NETs) are a diverse group of tumors that range from the indolent typically low grade gastroenteropancreatic neuroendocrine tumors (GEP-NET), thymic and lung NET to the rapidly growing, aggressive type such as small cell lung cancer [1]. Neuroendocrine tumors can arise as primary tumors in almost any organ system of the body including the gastrointestinal tract, pancreas, lungs, skin, endocrine glands, and prostate. The recent WHO classification separates GEP-NETs into three grades based on mitotic count and Ki-67 index (Table 1) [2]. The prognosis of those patients with localized NET is good, with 10 year survival rates of 68.2% reported in a large population-based series, however for patients with metastatic disease 10-year survival rates dropped to 17.5% [3].

**Table 1:** World Health Organization Grading for Neuroendocrine Tumor

GRADE	DIFFERENTIATION	GASTROENTEROPANCREATIC NETS	LUNG & THYMUS
LOW GRADE (G1)	Well-differentiated NET	<2 mitoses per 10 high-power field (HPF) AND/OR <3% Ki67 index	<2 per 10 HPF AND no necrosis
INTERMEDIATE GRADE (G2)	Well-differentiated NET	2-20 mitoses per 10 HPF AND/OR 3-20% Ki67 index	2-10 per 10 HPF AND/OR foci of necrosis
HIGH GRADE (G3)	Poorly differentiated neuroendocrine carcinoma	>20 per 10 HPF AND/OR >20% Ki67 index	>10 mitosis/10 HPF

Treatment options are limited and palliative in nature for patients with all types of metastatic NET. Systemic therapy with somatostatin analogues has been shown to provide control of carcinoid syndrome and improve quality of life in patients with NET, and two prospective randomized trials have shown improvement in progression-free survival compared to placebo, demonstrating the antiproliferative effects of these treatments; however tumor response rates are low [4–6]. Generally, liver-directed therapies including surgery, radiofrequency ablation (RFA) or transarterial chemoembolization (TACE) are offered to patients with metastatic or advanced disease with the goal of palliating symptoms or perhaps delaying liver failure and death [7]. Recently, the use of tyrosine-kinase inhibitor sunitinib and the mTOR-inhibitor everolimus resulted in a 5.9 month and 6.4 month improvement in progression free survival (PFS) when compared to placebo in pancreatic neuroendocrine tumors (pNET) [hazard ratio for death favoring sunitinib compared to placebo was 0.41 (95% CI, 0.19 to 0.89; P=0.02), and for everolimus 0.35 (95% CI, 0.27 to 0.45; P<0.001)] [8, 9]. Although stable disease rates were substantial in both trials, response rates were low: 5% for everolimus and 9.3% for sunitinib. Most recently, the RADIANT-4 trial assessed everolimus in low grade non-functional neuroendocrine tumors of lung or gastrointestinal origin [10]. RADIANT-4 was a phase 3, double-blind, placebo controlled trial that demonstrated a prolonged progression free survival in patients treated with everolimus compared to placebo (11.0 months compared to 3.9 months, hazard ratio 0.48, p<0.00001). In comparison, high dose dacarbazine had an overall response rate of 34% in a prospective single-agent trial for metastatic pNETs [11]. Temozolomide is an oral alkylating agent that was designed to be less toxic than dacarbazine and has been shown to be effective in patients with glioma and melanoma [12, 13]. Temozolomide was shown to be active in pancreatic neuroendocrine in combination with thalidomide [14] and bevacizumab [15] but was associated with significant toxicity including lymphopenia and serious opportunistic infections in 10% of patients in the thalidomide trial [14].

The synergistic activity of 5-FU and TMZ led to the development of temozolomide given in combination with capecitabine (CAPTEM). Capecitabine, which is converted to 5-FU, depletes tumor O^6^-methylguanine DNA methyltransferase (MGMT) levels thereby enhancing the alkylating effect of temozolomide. *In-vitro* studies showed that 5-FU depletes tumor levels of MGMT [16]. Synergistic activity was observed to be schedule-dependent, requiring TMZ to be given after continuous exposure to capecitabine [17]. Kulke et al studied MGMT levels by immunohistochemistry (IHC) in tumor tissue from 21 patients who were treated with temozolomide and capecitabine [18]. Response to treatment was seen in 80% (4/5) of patients with MGMT-deficient tumors while none of the 16 patients with intact MGMT expression had therapeutic response. Thus low MGMT expression seemed to correlate with response to treatment. However the role of MGMT as a predictor of response remains unclear in other studies [19, 20], and a larger retrospective study of 144 patients with pNET treated with CAPTEM found that neither MGMT deficiency nor Ki-67% predicted response to treatment [20]. CAPTEM has been evaluated in multiple trials, demonstrating impressive activity in grade 1 pancreatic neuroendocrine tumors with response rates up to 70% [17, 20–24]. The only phase II data for CAPTEM was presented at the 2014 ASCO session, and included 28 patients with metastatic grade 1 and 2 NETs including typical and atypical carcinoid tumors, pituitary, pancreatic NET, and medullary thyroid cancers, who progressed on octreotide. CAPTEM therapy was associated with 11% complete responses (CR), 32% partial responses (PR), and 54% stable disease (SD) for a disease control rate of 97%. Median PFS was over 20 months and median OS greater than 25.3 months at the time of interim analysis. The most common grade 3 or 4 adverse events were lymphopenia (32%), hyperglycemia (15%), thrombocytopenia (3%), and diarrhea (3%) [25]. Very little data exist for CAPTEM in non-pancreatic NET as well as intermediate and high-grade NET. A retrospective study of 29 patients demonstrated response rates in non-pancreatic NET vs pancreatic NET of 14% and 20%, respectively, with an overall response rate (ORR) of 17% and median PFS of 12 months [10].

**Figure 1:**
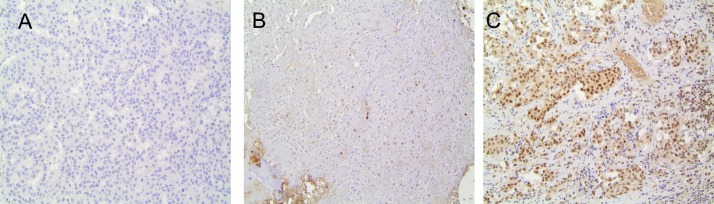
MGMT Expression by Immunohistochemistry (IHC) MGMT IHC was available for 20 patients. Representative slides are presented in the figure, demonstrating low **(A)**, intermediate **(B)**, and high **(C)** MGMT expression by IHC. Thresholds for expression were set as <10% for low, 10-49% for intermediate, and >50% for high MGMT expression.

In order to further clarify the efficacy and safety of CAPTEM in a wider population of NET patients than previously studied, and to evaluate MGMT IHC as a predictor of response (Figure 1), we report here a retrospective study of patients treated with CAPTEM therapy for advanced neuroendocrine carcinomas of all grades and both pancreatic and non-pancreatic origin.

## RESULTS

### Patient characteristics

Table 2 lists the characteristics of patients at the start of chemotherapy. Pancreas was the most common primary tumor location (61%) the followed by lung and rectum (8% each). Over one-half of patients (55%) previously received treatment with cytotoxic or targeted agents, and 3 patients (8%) received prior single agent temozolomide. Patients who received prior platinum based chemotherapy had Ki-67 ranging from 5-100%. Three patients (a patient with pNET with Ki-67 of 5%, a patient with NET of unknown primary with Ki-67 of 5-10%, and a patient with bronchial NET with Ki-67 of 10%) were treated with carboplatin and etoposide prior to being seen at our institution for a second opinion. The three patients who had previously been treated with single agent temozolomide tolerated it well and all had received last temozolomide dose between 411-484 days before treatment with CAPTEM. More than half of the patients (55%) had previously undergone at least one local therapy including surgical resection, trans-arterial chemo-embolization (TACE), radiofrequency ablation (RFA) or selective internal radiation therapy (SIRT). Seventeen patients (45%) were systemic therapy naïve and nearly a quarter of patients (24%) were treatment naïve with no prior regional or systemic therapy. One patient had previously received velcade and decadron for the treatment of multiple myeloma, but was not considered to have had prior systemic therapy for the purposes of this study.

**Table 2:** Patient Characteristics

	N	%
Total no. of patients	38	100%
**Median Age (range) years**	53 (22-84)	
**Gender**		
Male	22	58%
Female	16	42%
**Ethnicity**		
Caucasian	35	92%
African-American	3	8%
**NET Tumor Histology**		
Low grade NET (WHO 1, ki-67 < 3%)	10	28%
Intermediate Grade NET (WHO 2, ki67 3 - 20%)	16	46%
High Grade NET (WHO 3, ki67 > 20%)	9	26%
Pheochromocytoma	1	3%
Paraganglioma	1	3%
Grade Unknown	1	3%
**Primary Tumor Location**		
Pancreas	23	61%
Lung	3	8%
Rectum	3	8%
Stomach	1	3%
Adrenal	1	3%
Tonsil	1	3%
Ovary	1	3%
Thyroid	1	3%
Carotid Body	1	3%
Unknown	3	8%
**Prior Therapy**		
**Regional therapy**	21	55%
Surgery	14	37%
TACE/RFA/SIRT	12	32%
**No prior systemic therapy**	17	45%
**Prior Systemic therapy**	21	55%
Temozolomide alone	3	8%
Platinum Chemotherapy	10	26%
mTOR inhibitor	9	24%
**Prior Octreotide therapy**	15	39%

NET, neuroendocrine tumor. TACE, transarterial chemoembolization. RFA, radiofrequency ablation. SIRT, selective internal radiotherapy.

### Efficacy

**Figure 2:**
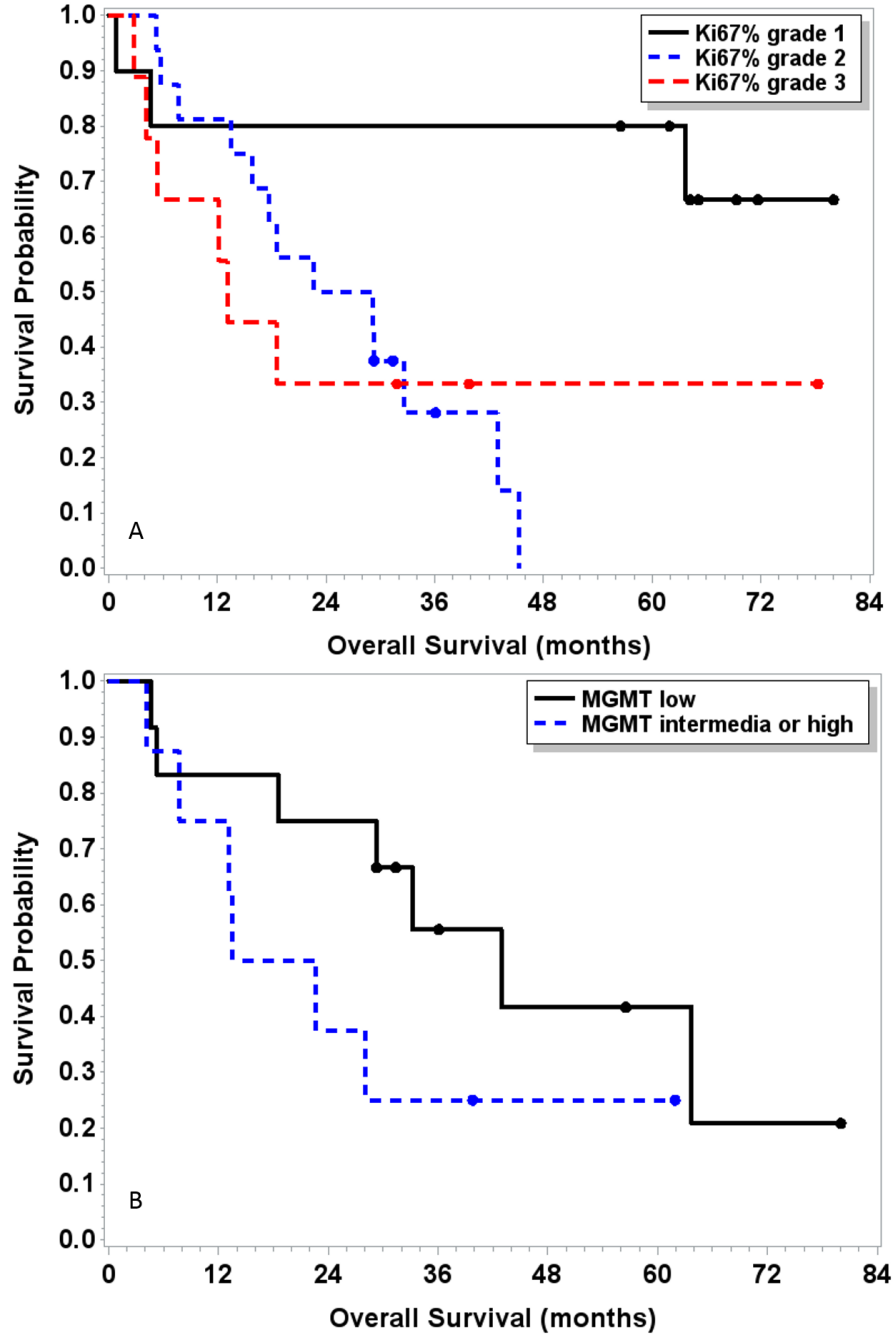
Kaplan Meier curves for survival by Ki-67% tumor grade **(A)** and MGMT **(B)**. Overall survival was not significantly associated with MGMT level (p=0.16), but significantly associated with Ki-67% grade (p=0.027). Survival rate at 2 years was higher in the MGMT low group (75%) compared to the MGMT intermediate-high group (38%) (p=0.08).

Among all patients, median PFS was 13.0 months and median overall survival (mOS) 29.3 months (Table 3, Figure 2). Patients with pNET had a significantly longer median PFS compared to non-pNET patients [16.7 months (95% CI: 6.1 – 41.9) versus 8.4 months (95% CI: 2.4 – 13.3, p=0.026] (Table 3). Systemic therapy naïve patients had a longer median PFS and median OS compared to those who had received prior systemic therapy [PFS: 17 months (95% CI 10.6 – NR) versus 6.1 months (95% CI 3.6 – 12.6), p=0.005; OS: 63.7 months (95% CI 28.1 – NR) vs 15.8 months (95% CI 5.8 – 32.6) p=0.008].

**Table 3:** Progression free survival and overall survival by MGMT expression and Ki-67%

	N	Median PFS (95% CI) (months)	p-value	Median OS (95% CI) (months)	p-value
All patients	38	13.0 (5.6-17.0)		29.3 (17.7 - 45.3)	
By MGMT expression:					
Low MGMT by IHC (<10%)	12	16.6 (4.5 – NR)	0.19	42.9 (5.2 – NR)	0.16
High MGMT by IHC (≥10%)	8	9.5 (3.4 – 21.3)		18.1 (4.1 – NR)	
By Ki-67%:					
Low grade (WHO 1)	10	20.0 (0.3 – NR)	0.34	NR (0.8 – NR)	0.027
Intermediate Grade (WHO 2)	16	9.5 (3.6 – 16.1)		25.9 (13.5 – 42.9)	
High Grade (WHO 3)	9	8.4 (1.2 – 37.9)		13.1 (2.8 – NR)	
By Tumor Origin Site					
Pancreas	23	16.7 (6.1 – 41.9)	0.026	42.9 (18.5 – NR)	0.12
Non-pancreas	15	8.4 (2.4 – 13.3)		18.5 (4.6 – 32.6)	

When stratified by proliferation index (Ki-67%), lower grade was associated with longer overall survival (Table 3). Median PFS was 20.0 months for patients with grade 1 tumors, 9.5 months for grade 2, and 8.4 months for grade 3. Median OS was not reached in patients with grade 1 NET, 25.9 months in grade 2 NET, and 13.1 months in patients with grade 3 NET. The difference was statistically significant for OS (p=0.027) but not PFS (p=0.34) across all grades.

**Figure 3:**
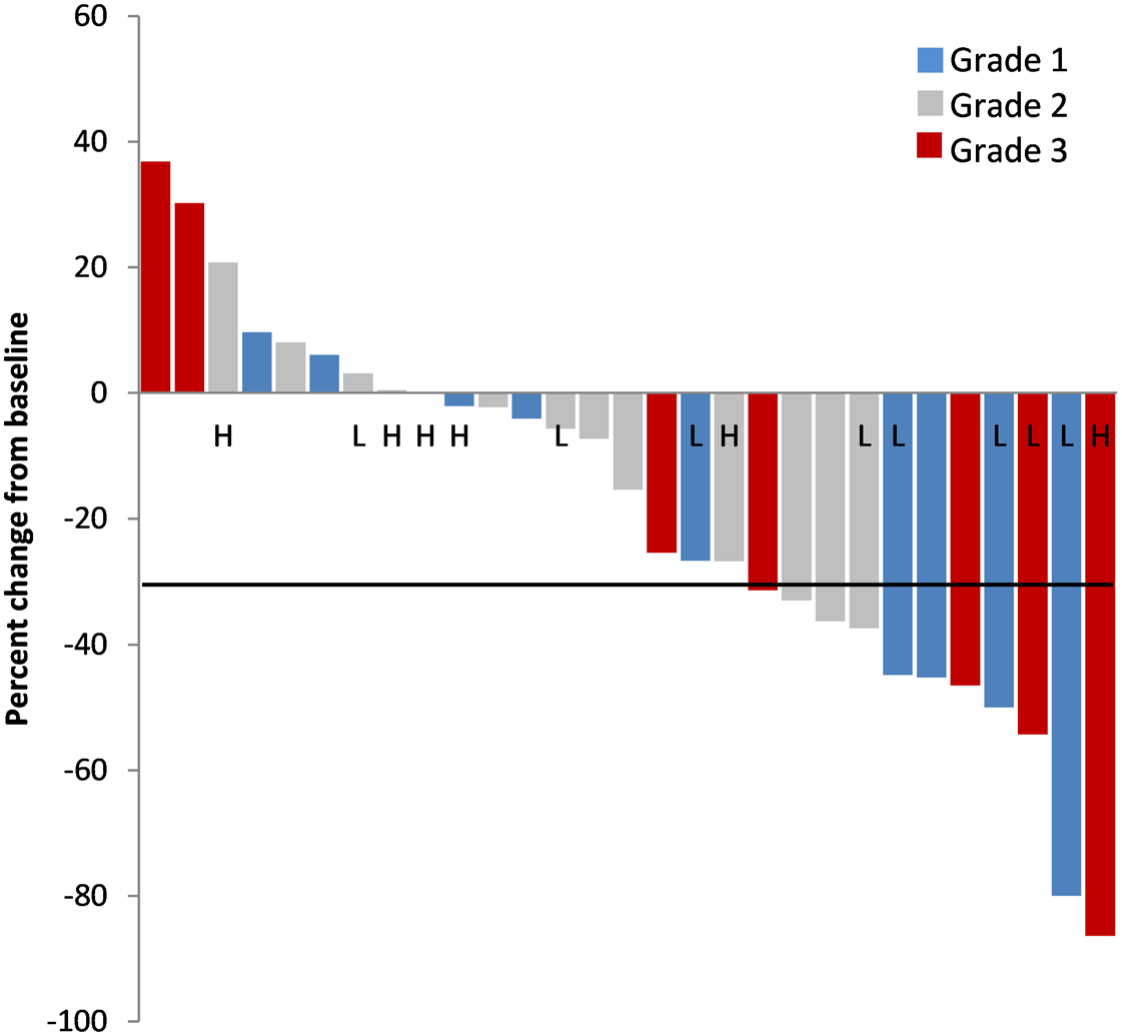
Waterfall plot Maximum percent change from baseline in the sum of the diameters of target lesions, by Ki-67% and MGMT IHC low (L) or intermediate/high (H). Patients with grade 1 tumors but unknown Ki-67% marked with (^*^).

All patients had imaging performed at baseline, but several patients did not have imaging performed at our institution during active treatment and were therefore excluded from response analysis. Response to treatment was assessed using RECIST v1.1 criteria, with 29 out of 38 patients having sufficient follow up to assess response to treatment, including 11 patients (38%) with partial response (PR), 15 patients (52%) with stable disease (SD), and 3 patients (10%) with progressive disease (PD), for a disease control rate of 90% (Table 4, Figure 3).

**Table 4:** Objective response rate by grade and MGMT expression

	N	PR	SD	PD	p-value
All patients	29	11 (38%)	15 (52%)	3 (10%)	
By MGMT expression:					
Low MGMT by IHC (<10%)	8	5 (63%)	3 (38%)	0 (0%)	0.18
Intermediate and High MGMT by IHC (≥10%)	6	1 (17%)	4 (67%)	1 (17%)	
By Ki-67%:					
WHO Grade 1	7	3 (43%)	4 (57%)	0 (0%)	0.19
WHO Grade 2	13	4 (31%)	8 (62%)	1 (8%)	
WHO Grade 3	7	4 (57%)	1 (14%)	2 (29%)	

For the 18 patients with pNET with follow-up data, there were 9 (50%) PR observed, 8 (44%) SD, and 1 PD (6%). For the 11 patients with NET not of pancreatic origin, there were 2 (18%) PR, 7 (64%) SD, and 2 (18%) PD, with no statistical difference in response between the two cohorts (p=0.21). Of the patients who had received prior systemic therapy, 16 patients could be evaluated, with a PR observed in 6 (38%), SD in 7 (44%), and PD in 3 (19%). All three patients previously treated with single-agent temozolomide achieved stable disease as best response. For the 13 systemic treatment-naïve patients who had follow-up imaging, PR was observed in 5 (38%), SD in 8 (62%), PD in 0. Of the six patients previously treated with either TKI or MTOR inhibition, 3 PR (50%), 2 SD (33%), and 1 PR (17%) were observed.

Thirty-five patients had Ki-67% reported with 10 classified grade 1 (Ki-67 < 3%), 16 as grade 2 (Ki67 3-20%), and 9 grade 3 (Ki67 ≥ 20%). Of the nine patients with grade 3 disease, one patient had well differentiated, two patients had moderately differentiated, and six patients had poorly-differentiated histology. Follow-up information was available for 7, 13, and 7 patients respectively. Of the seven patients with grade 1 tumors, PR were observed in 3 (43%), SD in 4 (57%), and no PD. For grade 2 tumors response rates included PR in 4 patients (31%), SD in 8 (62%), and PD in 1 (8%). For grade 3 tumors rates were PR in 4 (57%), SD in 1(14%), and PD in 2 patients (29%). Patients with grade 3 disease who achieved a clinical benefit from CAPTEM had Ki-67 indices of 20%, 25%, 35%, 40%, and 60%, whereas those that had PD were 50% and 100%.

Thirty patients had tumor markers within 30 days of starting treatment and periodically while on treatment (pancreastatin, gastrin, glucagon, neurotensin; metenephrines for pheochromocytoma/paraganlgioma). Of these, decrease of at least 50% in one elevated marker occurred in 17 patients (57%) while on therapy. Five patients received CAPTEM for a second time after stopping therapy initially due to achieving best response. Three patients were evaluable for response, 2 had stable disease as best response and 1 had progressive disease at CAPTEM re-challenge. Five patients were eligible for surgical resection following treatment with first-line CAPTEM.

Twenty patients had tumor tissue available for MGMT staining, of whom 12 had low and 8 had intermediate-high expression. Fourteen of 20 patients (6 intermediate-high and 8 low expression) had sufficient follow-up to assess response. Of the 8 patients with low MGMT expression by IHC and evaluable response there were 5 PR (63%), 3 SD (38%), and 0 PD. There was a trend toward longer PFS (median 16.6 months vs 9.5 months, p=0.19) and longer OS (42.9 months vs 18.1 months, p=0.16) for patients with low MGMT expression compared to those with high expression. Of the 6 patients with intermediate-high expression MGMT, 1 patient had PR (17%), 4 patients had SD (67%), 1 patient had PD (17%) as best response. Survival rate at 2 years was higher in the MGMT low group (75%) compared to the MGMT intermediate-high group (38%) (p=0.08).

Two patients with NET had mutations in *MEN1* gene, one patient with pheochromocytoma had a pathogenic *VHL* mutation in exon 3, and one patient with paraganglioma had a variant of uncertain significance in *SDHB* c.287-3C>G. One patient with *MEN1* gene: c.628_631delACAG mutation had grade 3 pancreatic NET (Ki-67 25%) with high MGMT expression, but still derived benefit from CAPTEM therapy with stable disease burden for 3 years after completion of 8 cycles. A second patient with *MEN1* G74fs^*^45 mutation had grade 1 pancreatic NET had a dramatic response to CAPTEM with 45% response in target lesions. One patient with lung carcinoid with Ki-67 < 5% without evidence of an inherited syndrome had somatic mutations detected including *PDGFRB* A366T and *CDKN1b* loss, and had best response of stable disease on treatment.

### Safety

All patients included in analysis received at least one cycle of therapy. Median number of cycles received was 4. Dose reductions or delays occurred in 15 patients (41%). Grade 3 or 4 adverse events were reported in 5 separate patients (14%), with two patients experiencing pancytopenia (Table 5). Five patients (13%) discontinued therapy due to adverse events, including thrombocytopenia (3 patients), bowel perforation (1 patient) and fatigue (1 patient). The most common grade 3 or 4 adverse events were thrombocytopenia (4 patients, 11%), leukopenia and anemia (2 patients, 5%), and nausea (1 patient, 3%). One patient suffered a Grade 4 bowel perforation while on treatment requiring urgent surgical intervention, with pathology revealing necrotic tumor invading the stomach. The most common side effect overall was fatigue in twenty patients (53%), none higher than grade 2. Other common grade 1 or 2 adverse events included nausea in 19 patients (50%), HFS in 5 patients (13%), anemia in 6 patients (16%), cognitive disturbance/memory impairment in 3 patients (8%), and diarrhea in 3 patients (8%). There were no treatment-related deaths.

**Table 5:** Adverse events associated with capecitabine and temozolomide combination therapy (CTCAE v 4.0)

Hematologic Toxicity	Grade 1	Grade 2	Grade 3	Grade 4
Anemia	2	4	2	0
Thrombocytopenia	4	3	0	4
Lymphopenia	2	4	0	0
Leukopenia	2	3	2	0
Neutropenia	1	1	1	0
Non-Hematologic Toxicity				
Fatigue	9	11	0	0
Nausea	14	5	1	0
Vomiting	2	2	0	0
Hand-foot syndrome	3	2	0	0
Mucositis	1	1	0	0
Ataxia	0	1	0	0
Confusion/Memory Impairment	2	1	0	0
Constipation	2	0	0	0
Diarrhea	3	0	0	0
Anorexia	1	3	0	0
Dysgeusia	2	0	0	0
Gastric Perforation	0	0	0	1*

*Surgical pathology revealed necrotic tumor invasion of the stomach.

## DISCUSSION

This study supports existing retrospective data for activity of capecitabine and temozolomide in pancreatic neuroendocrine tumors, and expands on existing knowledge of its role in non-pancreatic NET as well WHO grade 3 NET. In this study, we observed a disease control rate of 90% with combination therapy capecitabine and temozolomide, in patients with WHO grade 1, 2, or 3 neuroendocrine tumors in both treatment-naïve and pre-treated patients and in pNET and non-pNET. The median progression free survival observed of 13.0 months and median overall survival of 29.3 months compare favorably to prior studies [24]. The differences in overall survival seen in patients with grade 1 and grade 2 tumors (Table 3) should be interpreted with caution given the small sample size and large 95% confidence intervals. This difference may be due to differences in natural history of the disease and not the specific treatments received. This question will be better addressed by the clinical trial ECOG-E2211 (NCT01824875), a large prospective trial that has already met accrual and for which results are pending.

Prior retrospective studies have varied in terms of number of patients, dosing and scheduling of treatment regimens, histologies included, and overall low numbers of patients included with tumors other than pNET. Furthermore, there is limited and conflicting data regarding MGMT expression patterns and predictive value [26, 27]. Our data support the hypothesis that low MGMT expression correlates with favorable response to CAPTEM therapy, with a high PR rate (63%) than, and a trend toward longer observed PFS and OS when compared to intermediate-high MGMT protein expression. Interpretation of this finding is limited by the small number of patients whose tumors were available for MGMT expression testing. However, it should be noted that of the patients with intermediate-high expression of MGMT there was still one PR observed with 4 patients experiencing stable disease on treatment. This data supports that from other centers [28, 29], cautioning against the use of MGMT expression as the sole predictor of response to CAPTEM and further reinforcing the need for better biomarkers to aid in clinical decision making.

The activity of the regimen in patients treated previously with systemic therapies, including TKI and mTOR inhibition, as well as activity in grade 3 tumors bears further study in future prospective trials. Given that both sunitinib and everolimus have also been shown to be active in patients previously treated with chemotherapy [8, 9], further trials should focus on the optimal sequence and timing of CAPTEM and targeted therapies. The ability of patients to undergo palliative resection of tumors after CAPTEM therapy argues for further study in the use of this regimen as a neoadjuvant therapy. Additionally, the benefit seen in patients with Grade 3 NET with Ki67 ranging from 20-60% deserves further clinical study.

The outcomes observed in our study compare well with the results of a recent meta-analysis of temozolomide-based combination therapy for advanced neuroendocrine disease [26], where the reported median PFS ranged from 6 to 31 months and median OS from 22 to 83 months. The slightly higher rate of grade 3 or 4 adverse events rates seen in this population compared to CAPTEM given as first line therapy may be explained by the inclusion of heavily pre-treated patients and those with poorly differentiated disease in our cohort [22]. This study adds to the current knowledge base by including a large number of patients with a rare tumor, patients with higher grade tumors, patients treated previously with chemotherapy, everolimus and sunitinib, as well as the reporting of MGMT expression where tissue was available for analysis.

Limitations of our study include its retrospective nature, heterogeneous patient population, and limited follow-up data in some patients. As a regional subspecialty center, our referral base includes several neighboring states, and patients coming from long distances are typically co-managed with a local oncology team, which can limit the availability of follow-up radiologic data. Another limitation is the use of IHC staining to detect MGMT expression as opposed to methylation-specific polymerase chain reaction. Also, the lack of quantifiable information on patients’ symptoms while on chemotherapy limits our ability to determine how this regimen impacted patients’ quality of life.

In conclusion, the results of our retrospective review add further evidence to support the activity of CAPTEM in patients with advanced neuroendocrine carcinomas. Patients with low grade tumors, tumors of pancreatic origin, and tumors with low levels of MGMT expression seemed to have higher response rates. However responses were still observed in patients with high grade tumors, tumors with higher levels of MGMT expression, and in patients with tumors of nonpancreatic origin. Despite *in-vitro* support for synergistic activity of the CAPTEM regimen, this activity has not yet been confirmed in randomized prospective clinical trials. That hypothesis is currently being evaluated in ECOG-E2211 (NCT01824875), a trial comparing temozolomide to combination therapy with capecitabine in patients with advanced pNET. The final results of NCT00869050 are also soon expected to be available. Finally, although low levels of MGMT expression seem to correlate with better responses to CAPTEM, better biomarkers should be sought to assist in predicting which patients will respond to CAPTEM therapy.

## MATERIALS AND METHODS

A retrospective review of all patients with a diagnosis of metastatic neuroendocrine tumor who received treatment with combination capecitabine and temozolomide between June 1, 2009 and June 1, 2013 at the Ohio State University was performed. The study was approved by the Institutional Review Board at the Ohio State University. A total of 38 patients with any grade metastatic neuroendocrine tumors, medullary thyroid cancer, and pheochromocytoma/paraganglioma were included. Patients were included regardless of prior therapies, including prior chemotherapies and targeted therapies. Patients who had prior systemic therapy were initiated on CAPTEM due to clinical or radiological progressive disease, or intolerance to prior therapy. Newly diagnosed patients were initiated on therapy due to symptomatic disease or high tumor burden, including carcinoid symptoms (n=2) or development of ascites (n=2). Imaging was performed every 2-3 cycles. RECIST v1.1 criteria were used to assess object response to treatment. Temozolomide was dosed at 200 mg/m2/day on days 10-14 and capecitabine at 1500 mg/m2/day in two divided doses on days 1-14 on a 28-day cycle.

MGMT protein expression was evaluated using immunohistochemistry in patients who had tissue available in our neuroendocrine tumor tissue bank using MGMT murine antibody (Thermo Scientific, MS-470-B). Paraffin-embedded tissue was cut at 4-μm and sections were placed on positively-charged slides. Slides were then placed in a 60 degree Celsius oven for 1 hour, cooled, deparaffinized and rehydrated through xylene and graded ethanol solutions to water. All slides were quenched for 5 minutes in a 3% hydrogen peroxide aqueous solution to block for endogenous peroxidase. Slides were stained with the Intellipath Autostainer Immunostaining System. All incubations on the Autostainer were performed at room temperature. Slides were counterstained in Richard Allen hematoxylin, dehydrated through graded ethanol solutions, cleared with xylene, and cover-slipped. MGMT expression was determined based on percentage of tumor cells positive for nuclear staining, as per prior studies [30]. Low, intermediate, and high expression were defined by <10%, 10-49%, and ≥50% expression of MGMT protein respectively (Figure 2). Scoring was done in blinded fashion in respect to objective response/survival data.

### Statistical analysis

Patient characteristics were summarized using descriptive statistics. Categorical data were summarized as frequency and percentage and continuous variables as medians and ranges. Adverse events were also summarized by grade per the National Cancer Institute Common Terminology for Adverse Events (CTCAE), version 4 criteria using frequency and percentage. Progression free survival (PFS) was measured as time from the start of treatment with temozolomide and capecitabine to clinical or radiologic disease progression or death. Patients who remained alive without disease progression were censored at the date of last follow-up. Overall survival (OS) was calculated from the start of treatment with temozolomide and capecitabine to death from any cause. Patients who were still alive were censored at the date of last follow up. Survivals were estimated using the method of Kaplan-Meier and compared using log-rank test. P values < 0.05 were considered statistically significant. Statistical analysis was performed using SAS version 9.4 for Windows (SAS Institute, Cary, NC).

## References

[1] Cives M, Strosberg J (2014). An update on gastroenteropancreatic neuroendocrine tumors. Oncology (Williston Park).

[2] Rindi G, Kloppel G, Alhman H, Caplin M, Couvelard A, de Herder WW, Erikssson B, Falchetti A, Falconi M, Komminoth P, Korner M, Lopes JM, McNicol AM (2006). TNM staging of foregut (neuro) endocrine tumors: a consensus proposal including a grading system. Virchows Arch.

[3] Hallet J, Law CH, Cukier M, Saskin R, Liu N, Singh S (2015). Exploring the rising incidence of neuroendocrine tumors: a population-based analysis of epidemiology, metastatic presentation, and outcomes. Cancer.

[4] Kvols LK, Moertel CG, O'Connell MJ, Schutt AJ, Rubin J, Hahn RG (1986). Treatment of the malignant carcinoid syndrome. Evaluation of a long-acting somatostatin analogue. N Engl J Med.

[5] Caplin ME, Pavel M, Cwikla JB, Phan AT, Raderer M, Sedlackova E, Cadiot G, Wolin EM, Capdevila J, Wall L, Rindi G, Langley A, Martinez S (2014). Lanreotide in metastatic enteropancreatic neuroendocrine tumors. N Engl J Med.

[6] Rinke A, Muller HH, Schade-Brittinger C, Klose KJ, Barth P, Wied M, Mayer C, Aminossadati B, Pape UF, Blaker M, Harder J, Arnold C, Gress T (2009). Placebo-controlled, double-blind, prospective, randomized study on the effect of octreotide LAR in the control of tumor growth in patients with metastatic neuroendocrine midgut tumors: a report from the PROMID Study Group. J Clin Oncol.

[7] Bloomston M, Al-Saif O, Klemanski D, Pinzone JJ, Martin EW, Palmer B, Guy G, Khabiri H, Ellison EC, Shah MH (2007). Hepatic artery chemoembolization in 122 patients with metastatic carcinoid tumor: lessons learned. J Gastrointest Surg.

[8] Raymond E, Dahan L, Raoul JL, Bang YJ, Borbath I, Lombard-Bohas C, Valle J, Metrakos P, Smith D, Vinik A, Chen JS, Horsch D, Hammel P (2011). Sunitinib malate for the treatment of pancreatic neuroendocrine tumors. N Engl J Med.

[9] Yao JC, Shah MH, Ito T, Bohas CL, Wolin EM, Van Cutsem E, Hobday TJ, Okusaka T, Capdevila J, de Vries EG, Tomassetti P, Pavel ME, Hoosen S (2011). Everolimus for advanced pancreatic neuroendocrine tumors. N Engl J Med.

[10] Ramirez RA, Beyer DT, Chauhan A, Boudreaux JP, Wang YZ, Woltering EA (2016). The Role of Capecitabine/Temozolomide in Metastatic Neuroendocrine Tumors. Oncologist.

[11] Ramanathan RK, Cnaan A, Hahn RG, Carbone PP, Haller DG (2001). Phase II trial of dacarbazine (DTIC) in advanced pancreatic islet cell carcinoma. Study of the Eastern Cooperative Oncology Group-E6282. Ann Oncol.

[12] Middleton MR, Grob JJ, Aaronson N, Fierlbeck G, Tilgen W, Seiter S, Gore M, Aamdal S, Cebon J, Coates A, Dreno B, Henz M, Schadendorf D (2000). Randomized phase III study of temozolomide versus dacarbazine in the treatment of patients with advanced metastatic malignant melanoma. J Clin Oncol.

[13] Stupp R, Mason WP, van den Bent MJ, Weller M, Fisher B, Taphoorn MJ, Belanger K, Brandes AA, Marosi C, Bogdahn U, Curschmann J, Janzer RC, Ludwin SK (2005). Radiotherapy plus concomitant and adjuvant temozolomide for glioblastoma. N Engl J Med.

[14] Kulke MH, Stuart K, Enzinger PC, Ryan DP, Clark JW, Muzikansky A, Vincitore M, Michelini A, Fuchs CS (2006). Phase II study of temozolomide and thalidomide in patients with metastatic neuroendocrine tumors. J Clin Oncol.

[15] Chan JA, Stuart K, Earle CC, Clark JW, Bhargava P, Miksad R, Blaszkowsky L, Enzinger PC, Meyerhardt JA, Zheng H, Fuchs CS, Kulke MH (2012). Prospective study of bevacizumab plus temozolomide in patients with advanced neuroendocrine tumors. J Clin Oncol.

[16] Murakami J, Lee YJ, Kokeguchi S, Tsujigiwa H, Asaumi J, Nagatsuka H, Fukui K, Kuroda M, Tanaka N, Matsubara N (2007). Depletion of O6-methylguanine-DNA methyltransferase by O6-benzylguanine enhances 5-FU cytotoxicity in colon and oral cancer cell lines. Oncol Rep.

[17] Fine RL, Gulati AP, Krantz BA, Moss RA, Schreibman S, Tsushima DA, Mowatt KB, Dinnen RD, Mao Y, Stevens PD, Schrope B, Allendorf J, Lee JA (2013). Capecitabine and temozolomide (CAPTEM) for metastatic, well-differentiated neuroendocrine cancers: The Pancreas Center at Columbia University experience. Cancer Chemother Pharmacol.

[18] Kulke MH, Hornick JL, Frauenhoffer C, Hooshmand S, Ryan DP, Enzinger PC, Meyerhardt JA, Clark JW, Stuart K, Fuchs CS, Redston MS (2009). O6-methylguanine DNA methyltransferase deficiency and response to temozolomide-based therapy in patients with neuroendocrine tumors. Clin Cancer Res.

[19] Walter T, van Brakel B, Vercherat C, Hervieu V, Forestier J, Chayvialle JA, Molin Y, Lombard-Bohas C, Joly MO, Scoazec JY (2015). O6-Methylguanine-DNA methyltransferase status in neuroendocrine tumours: prognostic relevance and association with response to alkylating agents. Br J Cancer.

[20] Cives M, Ghayouri M, Morse B, Brelsford M, Black M, Rizzo A, Meeker A, Strosberg J (2016). Analysis of potential response predictors to capecitabine/temozolomide in metastatic pancreatic neuroendocrine tumors. Endocr Relat Cancer.

[21] Fine R, Fogelman D, Schreibman S (2005). Effective Treatment of Neuroendocrine Tumors with Capecitabine and Temozolomide. J Clin Oncol.

[22] Strosberg JR, Fine RL, Choi J, Nasir A, Coppola D, Chen DT, Helm J, Kvols L (2011). First-line chemotherapy with capecitabine and temozolomide in patients with metastatic pancreatic endocrine carcinomas. Cancer.

[23] Cros J, Hentic O, Rebours V, Zappa M, Gille N, Theou-Anton N, Vernerey D, Maire F, Levy P, Bedossa P, Paradis V, Hammel P, Ruszniewski P (2016). MGMT expression predicts response to temozolomide in pancreatic neuroendocrine tumors. Endocr Relat Cancer.

[24] Kotteas EA, Syrigos KN, Saif MW (2016). Profile of capecitabine/temozolomide combination in the treatment of well-differentiated neuroendocrine tumors. Onco Targets Ther.

[25] Fine RL, Gulati AP, Tsushima D, Mowatt KB, Oprescu A, Bruce JN, Chabot JA (2014). Prospective phase II study of capecitabine and temozolomide (CAPTEM) for progressive, moderately, and well-differentiated metastatic neuroendocrine tumors. J Clin Oncol.

[26] Abdel-Rahman O, Fouad M (2015). Temozolomide-based combination for advanced neuroendocrine neoplasms: a systematic review of the literature. Future Oncol.

[27] Koumarianou A, Kaltsas G, Kulke MH, Oberg K, Strosberg JR, Spada F, Galdy S, Barberis M, Fumagalli C, Berruti A, Fazio N (2015). Temozolomide in Advanced Neuroendocrine Neoplasms: Pharmacological and Clinical Aspects. Neuroendocrinology.

[28] Reidy DL, Basturk O, Kriplani A, Abou-Alfa GK, Jarnagin WR, Allen PJ, Saltz L, Klimstra DS, Zhang L (2014). MGMT immunohistochemistry (IHC) and exclusion of pancreatic NET (PanNET) patients from treatment with temozolomidebased therapy. J Clin Oncol.

[29] Strosberg JR, Cives M, Brelsford M, Black M, Meeker A, Ghayouri M. (2015). Identification of response predictors to capecitabine/temozolomide in metastatic pancreatic neuroendocrine tumors. J Clin Oncol.

[30] Ekeblad S, Sundin A, Janson ET, Welin S, Granberg D, Kindmark H, Dunder K, Kozlovacki G, Orlefors H, Sigurd M, Oberg K, Eriksson B, Skogseid B (2007). Temozolomide as monotherapy is effective in treatment of advanced malignant neuroendocrine tumors. Clin Cancer Res.

